# Identification of a prognostic model using cuproptosis-related genes in uveal melanoma

**DOI:** 10.3389/fcell.2022.973073

**Published:** 2022-08-30

**Authors:** Yao Chen, Xiaozhen Chen, Xianggui Wang

**Affiliations:** ^1^ Department of Ophthalmology, Xiangya Hospital, Central South University, Changsha, China; ^2^ Hunan Key Laboratory of Ophthalmology, Xiangya Hospital, Central South University, Changsha, China; ^3^ National Clinical Research Center for Geriatric Disorders, Xiangya Hospital, Central South University, Changsha, China; ^4^ Hunan Key Laboratory of Skin Cancer and Psoriasis, Xiangya Hospital, Central South University, Changsha, China

**Keywords:** uveal melanoma, cuproptosis, signature, prognosis, bioinfomatics

## Abstract

The most common intraocular malignancy in adults remains uveal melanoma (UVM), and those with metastatic disease have a poor outlook. Proliferation, angiogenesis, and metastasis of tumor cells can be triggered by cuproptosis, affecting the survival of cancer patients. Nonetheless, cuproptosis-related genes (CRGs) have not been identified in UVM. In this study, we analyzed 10 CRGs in 80 patients with UVM in the Cancer Genome Atlas (TCGA) database regarding the alterations of the genes including copy number variation and methylation. We further constructed a prognostic gene model using these CRGs and built the risk score formula. Univariate and multivariate Cox regression was applied to validate the risk score as an independent prognostic factor. The prognostic model was validated using 63 UVM samples from the GSE22138 cohort, an independent validation data set. Based on the risk scores for 80 patients with UVM from TCGA, we categorized the patients into high- and low-risk groups. Differentially expressed genes (DEGs) between groups were enriched in allograft rejection, hypoxia, glycolysis, TNFα signaling *via* NF-κB, and interferon-γ responses via Gene set enrichment analysis (GSEA). CD8 T cells and exhausted T cells were notably enriched in the high-risk group. In conclusion, the alteration of CRGs is related to patients with UVM, and the constructed CRG-related model may be helpful to predict the prognosis of such patients.

## Introduction

In adults, uveal melanoma (UVM) is the most common primary intraocular malignancy, occurring in approximately five people per million per year (Lamas et al., 2021). Although local ocular treatment including brachytherapy, proton beam therapy, and enucleation could somewhat limit tumor growth (Kapoor et al., 2016), life expectancy is limited once metastasis occurs. Approximately 80–90% of patients with UVM develop metastasis if left untreated (Stalhammar and Gill, 2022). Although diagnostic techniques and medical therapies have improved over the past decade, the rate of metastasis remains high at 50%, and most cases are fatal within 1 year (Smit et al., 2020). The detection of prognosis-related risk factors or molecular biomarkers would enable surgical removal of the premature metastasis and cautious follow-up (Carvajal et al., 2017; Dogrusoz et al., 2017), possibly leading to improvements in quality of life and survival.

The clinical predictors of poor prognosis in UVM include senescence, ciliary involvement, the mean diameter of the primary basal tumor mass, extraocular spread, and the epithelial pathological phenotype (Shields et al., 2012; Hamadeh et al., 2016). However, these factors play a limited role in individual therapy. UVM is resistant to conventional cytotoxic chemotherapy that inhibits pathways of the cell cycle or tumor cell metabolism and survival (Wei et al., 2022). As opposed to cutaneous melanoma, UVM is less susceptible to checkpoint inhibitors, and it carries distinct genetic mutations such as BAP1, GNAQ/11, SF3B1, and EIF1AX mutations, which function as prognostic factors for malignancy, and therapeutic targets (Field et al., 2018). Numerous clinical trials examined UVM on a molecular basis, targeting these mutations or mutation-involved pathways including the MAPK pathway, Hippo–YAP pathway, and PI3K pathway (Wei et al., 2022). Recently, tebentafusp, a T-cell redirecting bispecific fusion protein, has received its first approval by the FDA for the treatment of previously untreated HLA- A*02:01–positive adults with metastatic or unresectable UVM(Dhillon, 2022). However, the molecular mechanisms underlying UVM progression remain unsolved, it is imperative to identify key molecular targets for predicting cancer prognosis and treatment efficacy.

Cuproptosis, a form of copper-related mitochondrial cell death, is a recently discovered cell death modality, induced by either insufficient or excessive intracellular copper (Tang et al., 2022). Through lipoylation of mitochondrial proteins participating in the tricarboxylic acid (TCA) cycle and the subsequent accumulation of toxic lipoylated proteins, copper ignites cuproptotic cell death (Tsvetkov et al., 2022). Increased copper levels have been reported in several malignancies, and they trigger tumor cell proliferation, angiogenesis, and metastasis (Oliveri, 2022). Rational copper toxicity would have a beneficial effect on tumors relying on energy produced via oxidative phosphorylation (Kahlson and Dixon, 2022). UVM was reported to be highly dependent on oxidative phosphorylation, but research has not examined cuproptosis in UVM (Chattopadhyay et al., 2019).

Here, we carried out a bioinformatic analysis to discover prognostic cuproptosis-related genes (CRGs) in UVM and analyzed the gene expression, immune infiltration, and prognosis to clarify the roles of these genes as biomarkers in cancer development as well as potential therapeutic targets.

## Methods

### Training and validation cohorts

All mRNA expression profile and relevant clinical data of the 80 patients with UVM were obtained from The Cancer Genome Atlas (TCGA) database as the training cohort (https://portal.gdc.cancer. gov/). For the independent validation cohort, mRNA expression datasets of 63 UVM samples (GSE22138) were obtained from Gene Expression Omnibus (GEO). Relevant guidelines and regulations were followed for all methods. Duplicates and cases with missing clinical outcomes were removed. We used Perl to convert Ensembl ID numbers into gene symbols based on the Ensembl database.

### Defining cuproptosis-related genes

CRGs were extracted from prior studies, as presented in Supplementary File S1 (Tang et al., 2022; Tsvetkov et al., 2022).

### Alterations of cuproptosis-related genes in uveal melanoma

The alterations of CRGs in the training cohort (including changes in genetic expression, copy number variation [CNV], and status of methylation) were examined on the Gene Set Cancer Analysis website (http://bioinfo.life.hust.edu.cn/GSCA/#/) (Liu et al., 2018). We studied the effect of alteration of CRGs on disease special survival (DSS), overall survival (OS) and progression free survival (PFS) of the patients.

### Construction of a prognostic signature with cuproptosis-related genes

In the training cohort, a least absolute shrinkage and selection operator (LASSO) penalty was calculated using the “glmnet” package in R software to create the best prognostic signature with the fewest RNA processing factors. A tenfold cross-validation was conducted to select the best penalty parameter (*λ*) and derive regression coefficients for independent variables. The formula is presented as: risk score = $\Sigma_{\text{i}=1}^{n}$ Coef_i_ x E_i_ (Each gene’s normalized expression value is Ei and Coefi is the coefficient). The hazard ratio was measured based on univariate and multivariate Cox regression. The forest maps for univariate and multivariate Cox analysis were drawn via the “forestplot” package in R.

### Evaluation of the cuproptosis-related genes-related prognostic model

In the training and validation cohorts, a median risk score was used to categorize patients to high- or low-risk groups. Risk scores were tested for prognostic value by comparing the survivability within groups. Kaplan Meier curves were generated using the “survival” package in R. We applied a heat map to illustrate the differences in expression of CRGs between the groups using the “pheatmap” package in R. Using the “survminer” and “timeROC” packages in R, time-dependent receiver operating characteristic curve (ROC) analysis was used to determine the specificity and sensitivity of the prognostic signature over the 1-,3-, and 5-year period. The area under the ROC curve (AUC) represented the accuracy of prognostication. The “rms” package in R was used to build a nomogram that predicts the survival of uveal melanoma patients (3-, 5-year survival) integrating the age, pathologic stage, T-stage, gender, and risk score.

### Functional analysis

After obtaining the COUNT data of the 80 samples from TCGA, DEGs within high- and low-risk groups in the training cohort were screened using the “DESeq2” package in R according to the following criteria: adjusted *p* < 0.05 and |log2Fold change| > 1. The volcano plot was charted using the “ggplot2” package in R, presenting the results of DEGs in the low-risk group versus the high-risk group. The ClusterProfiler package in R (version 3.18.0) was applied to explore the Gene Ontology (GO) functions and enrich the Kyoto Encyclopedia of Genes and Genomes (KEGG) pathways of possible targets. The org.Hs.eg.db R package (version 3.10.0) was employed for ID transformation. The DEGs were subsequently studied within the gene sets of c7.all.v7.5.1.symbols and hallmark genes of h.all.v7.5.1.symbols through gene set enrichment analysis (GSEA) (software 4.1) via a Java program. The gene sets were obtained from MSigDB Collections (https://www.gsea-msigdb.org/gsea/msigdb/collections.jsp#C2). The random sample permutation number was set at 1,000, and the significance threshold was set at *p* < 0.05, with a false discovery rate (FDR) < 0.25.

### The infiltration of immune cells

Our study compared immune cell infiltration of 24 immune cells between high- and low-risk groups using Immune Cell Abundance Identifier (ImmuCellAI) (http://bioinfo.life.hust.edu.cn/web/ImmuCellAI/) (Miao et al., 2020). We divided the 80 patients with UVM into high- and low-risk groups according to their median risk scores, and uploaded the expression file to the website to explore immune cell abundance and infiltration.

### Cell culture

We cultured UVM cell line M619, which was obtained from BeNa Culture Collection (Hebei, China). UVM cells were cultured in RPMI 1640 (Invitrogen, Carlsbad, CA, United States) with 10% fetal bovine serum (FBS, ExCell Bio) at 37°C in a 5% CO_2_ humidified incubator. The STR cell authentication report was obtained from the supplier. The Medical Ethics Committee of Xiangya Hospital, affiliated with Central South University, approved the cell experiments.

### Cell viability assay

Elesclomol (MedChemexpress Co., LTD. # HY-12040) with Copper chloride dihydrate (CuCl_2_, Sigma-Aldrich, # C3279) was used as the inducer of cuproptosis (Tsvetkov et al., 2019). Tetrathiomolybdate (TTM) (Sigma-Aldrich. #323446) was used as the inhibitor of cuproptosis (Tsvetkov et al., 2022). 5×10^3^ cells in suspension were seeded on a 96-well plate and allowed to adhere overnight in the culture medium containing 10% FBS. The cells were initially treated with or without 5 μM TTM overnight. Pre-mix CuCl_2_ and elesclomol with 1:1 ratio (elesclomol- CuCl_2_). Subsequently, the cells were treated with different concentration of the elesclomol- CuCl_2_ (0, 12.5, 25, 50, 100, and 200 nm respectively) for 24 h. Cell viability assay was performed using Cell Counting kit-8 (CCK8, Selleck, Shanghai, China) according to the manufacturer’s instructions.

### Cell invasion assay

Transwell cell culture systems with 8 μM pores in the upper chamber membrane were used for cell invasion assays. Matrigel (Corning, NY, United States) was diluted (1:7) in serum-free RPMI 1640 and was applied to the upper chamber membrane. 1 cells × 10^4^ cells in suspension were seeded on each upper chamber and cultured in a serum-free medium with 0 nm or 12.5 nm of elesclomol- CuCl_2_. The lower chamber was filled with RPMI 1640 medium containing 30% FBS. After culturing for 24 h at 37°C and 5% CO_2_, the cells were fixed with 4% formaldehyde and stained with 0.1% crystal violet. By wiping the cotton swab with 70% ethanol solution, all cells on the upper side of the membrane were removed. An inverted microscope (Ti-S, Nikon, Tokyo, Japan) was used to picture the invaded cells on the backside of the membrane. ImageJ software was used to count the number of invaded cells. The mean value for each field of view was calculated by randomly selecting three fields of view at 200-fold magnification.

### Scratch wound-healing migration assay

The UVM cells were seeded on a 6 well plate (5 cells × 10^5^ cells per well) and incubated under standard conditions overnight until the cells reached confluence. A straight scratch was created using a 10P pipette tip. The cells were treated with 0 and 12.5 nm of elesclomol- CuCl_2_ respectively. Photomicrographs were taken at 0 and 24 h. Images were captured using an inverted light microscope (Ti-S, Nikon, Tokyo, Japan). ImageJ software was used to measure the area of wound. A wound closure rate was calculated as follows: (original wound area - unhealed wound area)/original wound area ×100%.

### Statistical analysis

Student t test was used for comparison between two groups. Comparisons between multiple groups applied Wilcoxon test. Log-rank testing was used to determine the significance of the differences in survival. *p* < 0.05 was considered statistically significant.

## Results

### The expression, opy number variation and methylation of cuproptosis-related genes in uveal melanoma

**FIGURE 1:**
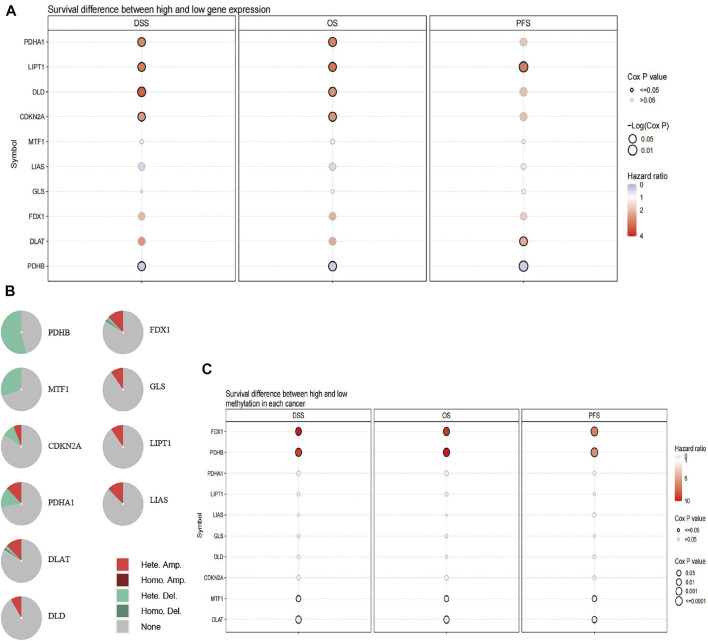
Alteration of CRGs in patients with UVM. **(A)** Patient survival differs between those with high and low expression of CRGs. **(B)** The difference of survival between patients with genes with CNV and those with wild-type genes. **(C)** The difference in survival according to the methylation status of CRGs in patients with UVM.

We studied the expression of CRGs in patients with UVM using TCGA cohort data and found that high expression of pyruvate dehydrogenase complex E1 alpha subunit (PDHA1), lipoyltransferase 1 (LIPT1), dihydrolipoamide dehydrogenase (DLD), and cyclin-dependent kinase inhibitor 2A (CDKN2A) and low expression of pyruvate dehydrogenase complex E1 beta subunit (PDHB) were associated with short disease-specific survival (DSS) and OS, whereas high expression of LIPT1 and DLAT and low expression of PHDB were related to short progression-free survival (PFS, Figure 1A). The percentage of CRGs with CNV is presented in Figure 1B. Heterozygous deletion of PDHB, metal-regulatory transcription factor 1 (MTF1), CDKN2A, and PHDA1 was noted, and heterozygous amplification was revealed for PDHA1, DLAT, DLD, FDX1, glutaminase (GLS), LIPT1, and lipoic acid synthase (LIAS). High methylation of FDX1 and PDHB were linked to poor DSS, OS, and PFS with hazard ratio above 1, whereas low methylation of MTF1 and DLAT were related to short DSS, OS, and PFS with hazard ratio less than 1 (Figure 1C).

### Building a signature related tocuproptosis-related gene in the training cohort

**FIGURE 2:**
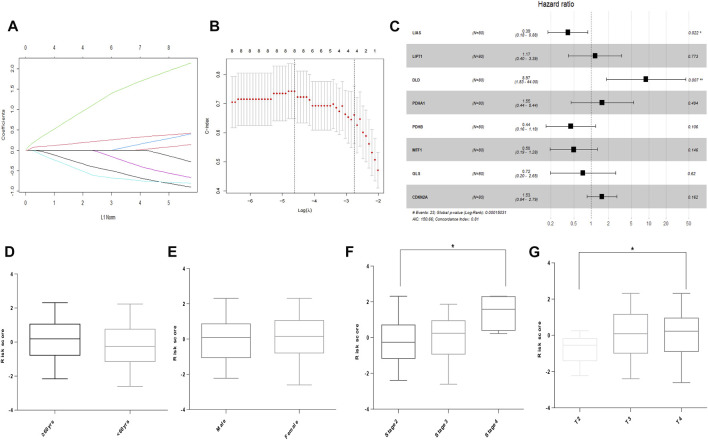
Building of the prognostic signature in UVM. **(A)** CRG profiles based on LASSO coefficients. **(B)** LASSO coefficient values of the CRGs in UVM. The vertical dashed lines are the optimal logλ values. **(C)** Hazard ratio, confidence interval, and *p*-value of the eight prognostic CRGs in univariate Cox regression. **(D)** Risk score in patients by age using a cutoff of 60 years. **(E)** Risk score in men and women. **(F)** Risk score in patients with different pathologic stages. **(G)** Risk score of patients with different T stages. * denotes *p* < 0.05.

In the TCGA cohort, LASSO was used to develop an optimal prognostic signature with a minimum number of RNA processing factors (Figures 2A,B). The formula was: risk score = (−0.764) × LIAS + (0.0503) × LIPT1 + (1.847) × DLD + (0.230) × PDHA1 + (−0.776) × PDHB + (−0.501) × MTF1 + (−0.067) × GLS + (0.355) × CDKN2A. Univariate Cox regression was applied to measure the hazard ratio of the eight selected genes, and the result illustrated that LIAS and DLD had prognostic value (Figure 2C). Next, we explored whether the risk score was related to clinical factors. The risk score did not differ according to patient age using a cutoff of 60 years (*p* = 0.1747, Figure 2D). Risk scores for men and women were not different (*p* = 0.8672, Figure 2E). Patients with pathologic stage 4 tumors were scored higher than those with stage 2 tumors (*p* = 0.0207, Figure 2F). Moreover, the risk score was higher in patients with T-stage 4 than T-stage 2 tumors (*p* = 0.0365, Figure 2G).

**FIGURE 3:**
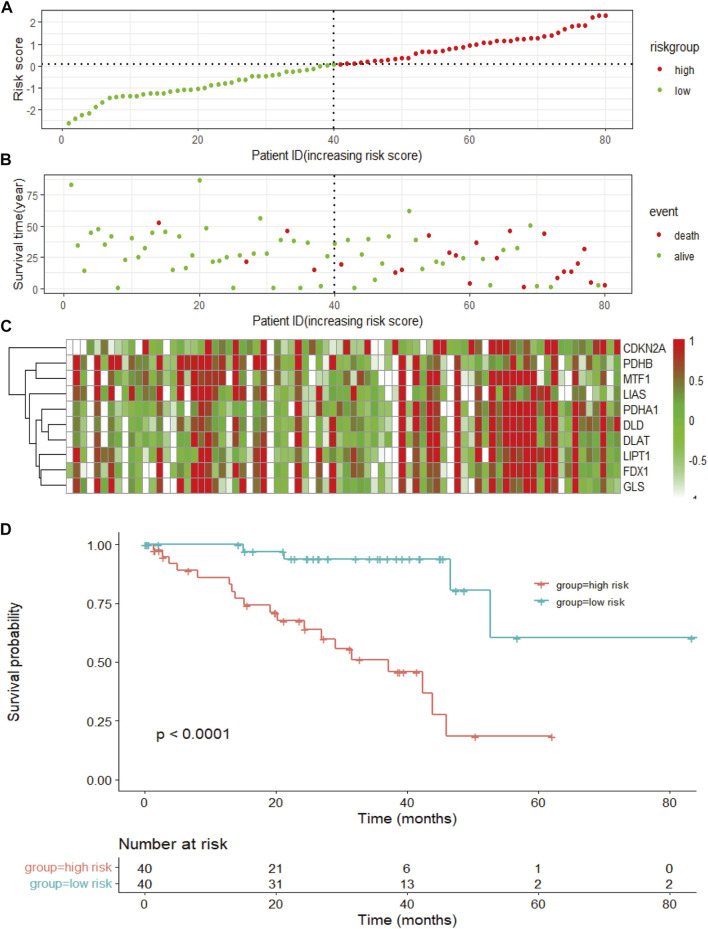
The risk score of the signature and survival in the TCGA cohort. **(A)** The risk score, survival time, and survival status of patients with UVM. **(B)** The scatter plot distribution represents the risk score of patients with UVM corresponding to the survival time and survival status. **(C)** The heatmap presents the expression of the eight selected CRGs in patients with UVM. **(D)** Kaplan-Meier survival analysis using the log-rank test for comparison of different groups within the risk model.

**FIGURE 4:**
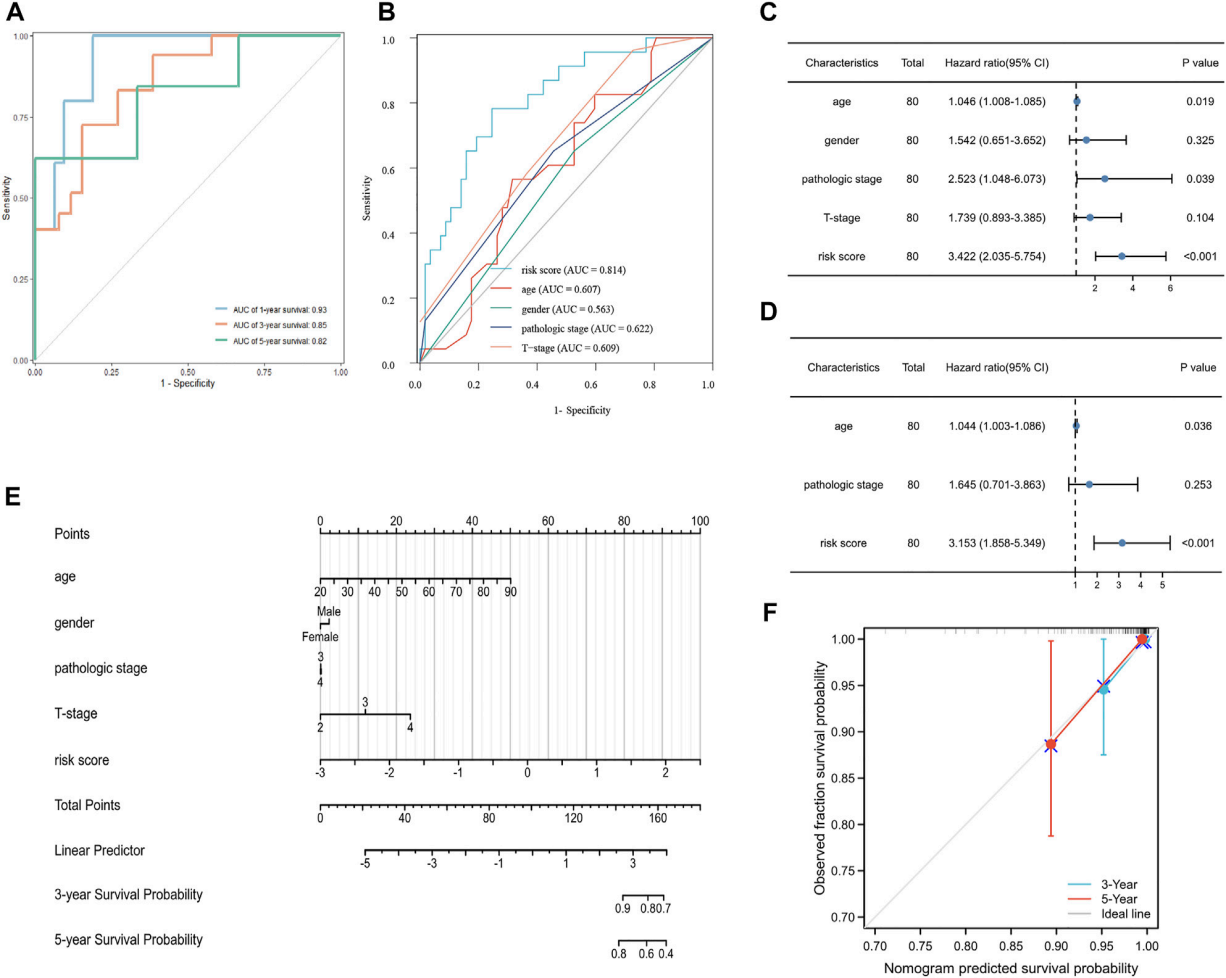
Validation of the prognostic signature in UVM. **(A)** The ROC curve and AUC of the signature at 1, 3, and 5 years. **(B)** The ROC curve and AUC for the risk score, age, gender, pathologic stage, and T stage. **(C)** Univariate Cox analysis for the risk score, age, gender, pathologic stage, and T stage. **(D)** Multivariate Cox analysis for the risk score, age, and pathologic stage. **(E)** Nomogram to predict the 3-, and 5-year OS of patients with UVM. **(F)** Calibration plot of the nomogram at 3, and 5 years.

To measure the prognostic value, we divided the patients into high- and low-risk groups based on the median risk score of the signature. Risk scores elevated with increasing deaths (Figure 3A). The heatmap revealed the expression of the CRGs in TCGA cohort (Figure 3B). Compared to the high-risk group, OS was longer in the low-risk group (*p* < 0.0001, Figure 3C). Besides, the AUCs of the 1-, 3-, and 5-year ROC curves were 0.93, 0.85, and 0.82, respectively (Figure 4A). The subsequently calculated AUCs for age, gender, pathologic stage, T-stage, and risk score were 0.814, 0.607, 0.563, 0.622, and 0.609, respectively (Figure 4B). We applied univariate (Figure 4C) and multivariate Cox (Figure 4D) analysis to clinic pathological factors together with risk score. The result of Cox analysis revealed age and risk score were independent prognostic factors for patients with UVM. The clinical factors such as age, gender, pathologic stage, T-stage, and the risk score were integrated into a nomogram for OS prediction at 3 and 5 years (Figure 4E), with C-index as 0.854 (*p* < 0.0001; 95% confidence interval = 0.815–0.893). The calibration plot suggested consistency between the observation and prediction (Figure 4F).

### The CRG-related signature in an independent validation cohort

**FIGURE 5:**
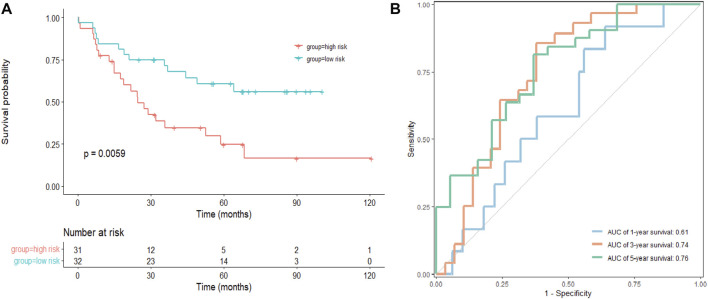
Validation of the signature in a GEO cohort. **(A)** Kaplan–Meier survival analysis of the risk model in GSE22138, and comparisons among different groups were performed using the log-rank test. **(B)** The ROC curve and AUC of the signatures of 1-, 3- and 5-year survival.

As a next step, an independent validation cohort from GEO (GSE22138) was downloaded to test the prognostic value of the model. We divided patients into low- and high-risk groups with the median risk score as cutoff. The Kaplan–Meier survival curve demonstrated that patients in the low-risk group had longer OS comparing to those in the high-risk group (Figure 5A). Time-dependent ROC curves showed that the AUCs were 0.61, 0.74, and 0.76 for 1-, 3-, and 5-year OS, respectively (Figure 5B), suggesting that the signature had potential for predicting OS in patients with UVM.

### Functional analysis

**FIGURE 6:**
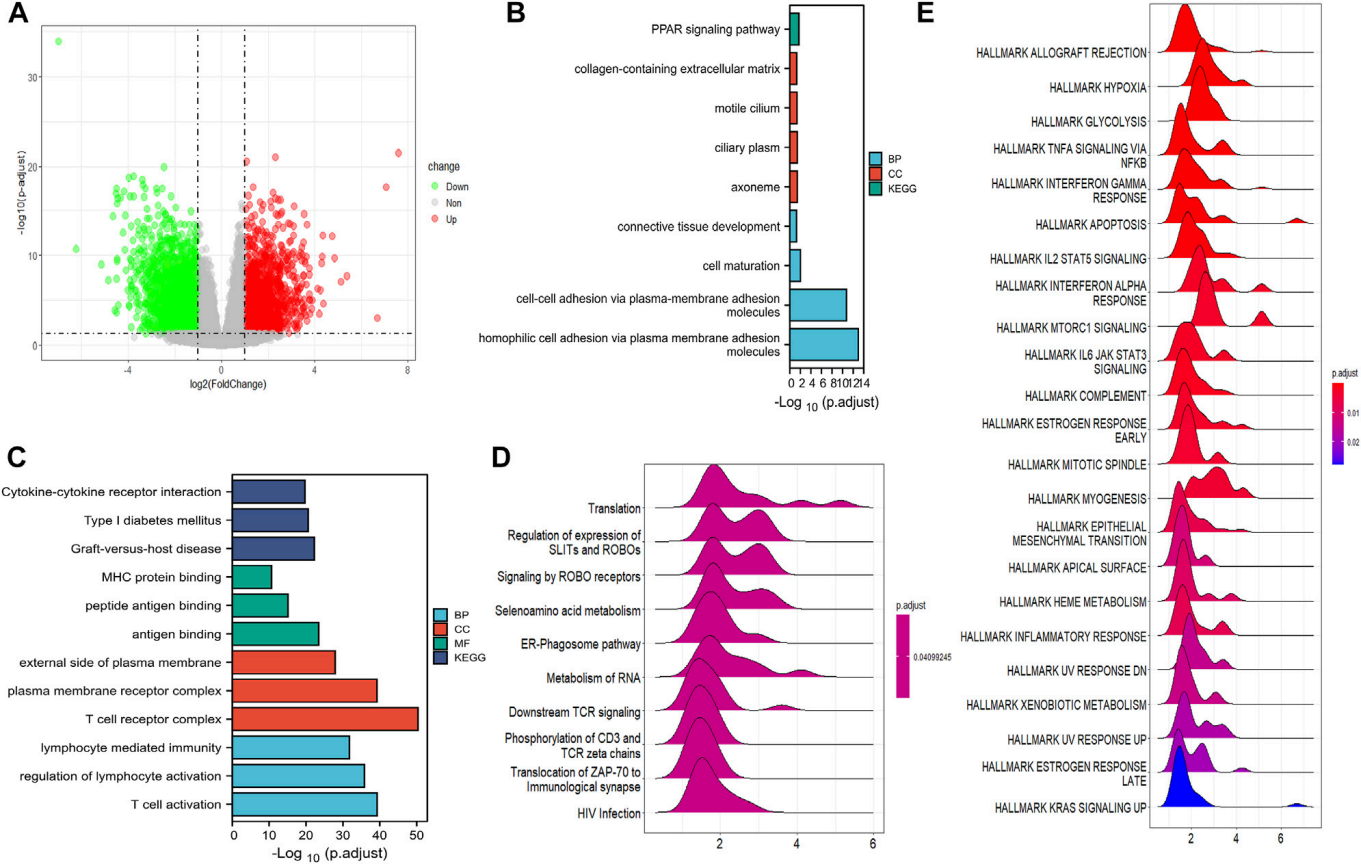
Function analysis of DEGs. **(A)** The volcano plot depicts the genes with differential expression between the high- and low-risk groups in TCGA cohort. **(B)** GO and KEGG analysis of the downregulated CRGs. **(C)** GO and KEGG analyses of the upregulated DEGs. **(D)** GSEA results of DEGs in GO datasets. **(E)** The DEG enrichment in the hallmark gene set by GSEA analysis.

Genes with differential expression between low- and high-risk groups in the training cohort were visualized using volcano plots (Figure 6A). In total, 4,088 DEGs were found with adjusted *p* < 0.05 and |log2Fold change| > 1. GO and KEGG pathway analyses demonstrated that the downregulated DEGs were predominantly involved in cytokine receptor interactions, immune cell activation, and protein binding (Figure 6C). The upregulated DEGs were mostly involved in cell–cell adhesion, extracellular matrix, cilium-based motility, and the peroxisome proliferator-activated receptor (PPAR) signaling pathway (Figure 6B). Furthermore, GSEA revealed that DEGs were primarily enriched in translation, regulation of SLIT and ROBO expression, and ROBO receptor signaling (Figure 6D).

### Immune infiltration patterns

**FIGURE 7:**
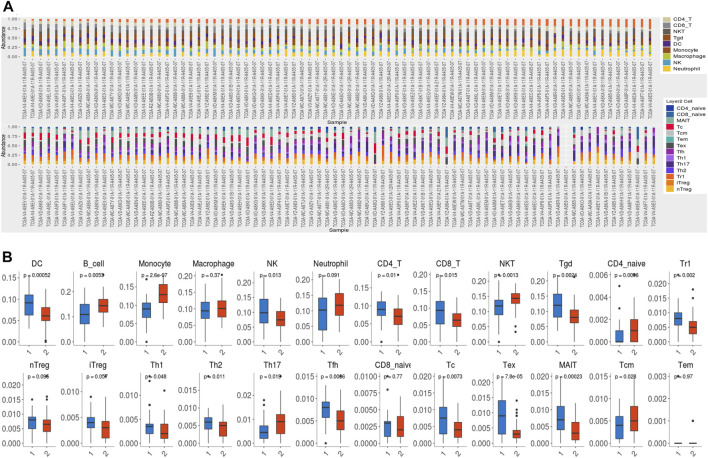
Immune infiltration. **(A)** TCGA cohort samples of 80 UVM samples contained 24 immune cell types. **(B)**The difference in immune cell infiltration between the high- and low-risk groups. DC: dendritic cells; NK: natural killer cells; NKT: natural killer T cell; Tgd: γδT cells; Tr1: type 1 regulatory T cells; nTreg: natural regulatory T cells; iTreg: induced regulatory T cells; Th1: T helper cell type 1; Th2: T helper cell type 2; Th17: T helper cell type 17; Tfh: follicular helper T cells; Tc: cytotoxic T cells; Tex: exhausted T cells; MAIT: mucosal associated invariant T cells; Tcm: central memory T cells; Tem: effector memory T cells.

We subsequently investigated the enrichment of DEGs in the hallmarks gene set by GSEA. The top five hallmarks were allograft rejection, hypoxia, glycolysis, TNFα signaling *via* the NF-κB pathway, and interferon-γ responses (Figure 7A). Moreover, an analysis of the immune infiltration was performed between the low- and high-risk groups in the training cohort. Immune cells such as dendritic cells (DC), natural killer (NK) cells, Tr1 cells, monocytes, follicular helper T (Tfh) cells, γδT cells, cytotoxic T (Tc) cells, mucosal-associated invariant T (MAIT) cells, exhausted T (Tex), CD8 T cells and CD4 T cells were notably found at higher levels in patients in the high-risk group than in those in the low-risk group, whereas B cells, monocytes, natural killer T (NKT) cells, CD4 naïve T cells, T helper cell type 17 (Th17) cells, and central memory T (Tcm) cells were enriched in the low-risk group (Figure 7B). However, no significant difference was found in the total infiltration scores between high- and low-risk groups (*p* = 0.12). We predicted the response to immune checkpoint blockade (ICB) therapy among patients with UVM via ImmuCellAI and found that only 1 patient would benefit from ICB therapy (Supplementary File S2).

### The effect of cuproptosis on viability, migration, and invasiveness of UVM cells

**FIGURE 8:**
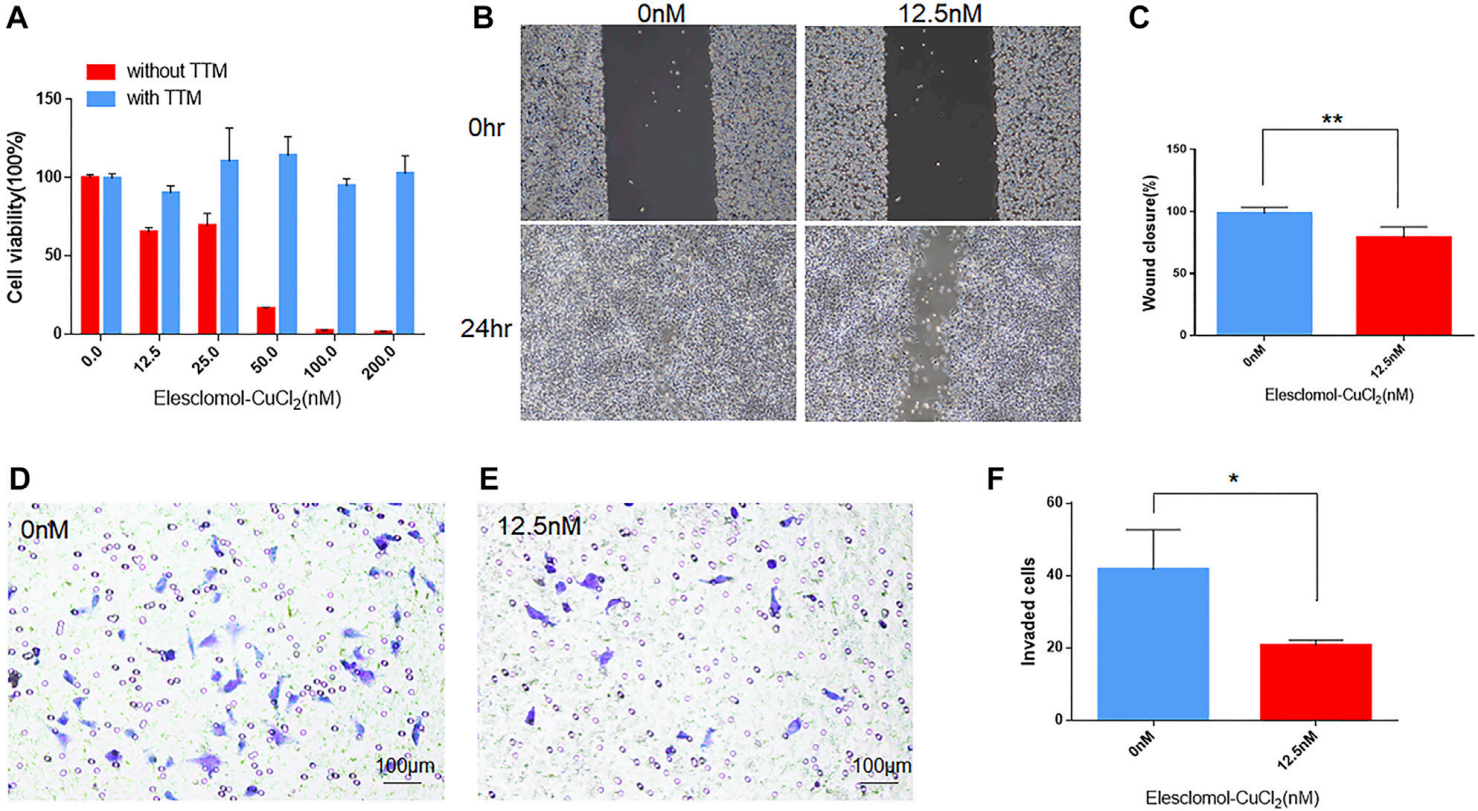
The effect of cuproptosis on UVM cells. **(A)** In cell viability assay, blue bars indicate cells treated with cuproptosis inhibitor TTM, red bars indicate cells treated without TTM. With the subsequent increasing concentration of cuproptosis inducer elesclomol-CuCl_2_ (elesclomol mixed with CuCl_2_ at 1:1 ratio), cell viability decreased. **(B)** In migration assay, the left panels showed the wound width of M619 cells without elesclomol-CuCl_2_ at 0 and 24 h, the right panels showed the wound width of M619 cells with 12.5 nm elesclomol-CuCl_2_ at 0 h and 24 h. **(C)** Wound closure rate is compared between cells with 0 and 12.5 nm elesclomol-CuCl_2_ at 24 h. **(D)** In transwell invasion assay, representative images of the penetrated M619 cells treated with 0 nm elesclomol-CuCl_2_. **(E)** Representative images of the penetrated M619 cells treated with 12.5 nm elesclomol-CuCl_2_. **(F)** Comparison of the invaded cells treated with 0 and 12.5 nm elesclomol-CuCl_2_.**p value<0.01; *p value<0.05.

To investigate the effect of cuproptosis on viability, migration, and invasiveness of UVM cells, we carried out cell viability assay, migration assay, and transwell invasion assay with cuproptosis inducer (elesclomol- CuCl_2_) or inhibitor (TTM). In cell viability assay, the inhibition of cell viability decreased steadily alongside increasing elesclomol- CuCl_2_ concentrations (Figure 8A). In addition, the inhibitive effect on viability in UVM cells could be reversed by the treatment of cuproptosis inhibitor TTM (Figure 8A), indicating the pivotal role of cuproptosis in cell viability of UVM. In cell migration assays, after treating with 12.5 nm elesclomol- CuCl_2_ for 24 h, the ability of migration was inhibited in M619 cells (Figure 8B), with the wound closure rate significantly decreased (*p* = 0.0076, Figure 8C). In cell invasion assay, transwell added with 12.5 nm elesclomol- CuCl_2_ showed less invaded cells (Figure 8E) compared to 0 nm elesclomol- CuCl_2_ (Figure 8D), *p* = 0.0304 (Figure 8F). These results confirmed that cuproptosis influences migration and invasion in UVM cells.

## Discussion

Copper accumulation in the cell leads to lipoylated proteins aggregating and striking Fe–S cluster proteins, resulting in cuproptosis, a distinct pattern of cell death (Tang et al., 2022), which is associated with copper transportation, and copper homeostasis (Cobine et al., 2021), and copper-related cell death (Zischka and Kroemer, 2020). Copper contributes to cancer development, as well as proliferation and metastasis of cancer cells (Oliveri, 2022). Cuprous oxide nanoparticles were demonstrated to impair cancer cell viability (Vegas et al., 2021), migration, and invasion in patients with UVM(Song et al., 2015). However, no prior studies examined the role of CRGs in UVM, prompting this study.

First, we analyzed the changes of CRG expression in patients with UVM in TCGA cohort because changes in gene expression and methylation are related to patient survival. Moreover, CNV was detected in all CRGs, and we considered that the CRGs might be able to predict patients’ prognoses with UVM. We subsequently performed LASSO regression analysis and selected eight CRGs (LIAS, LIPT1, DLD, PDHA1, PDHB, MTF1, GLS, and CDKN2A) to build a prognostic gene model. LIAS codes for constituents of the lipoic acid pathway and generates the antioxidant α-lipoic acid (LA) in the mitochondria (Dorsam and Fahrer, 2016). LIPT1 is a critical upregulated gene in LA pathways that mediates copper-induced cell death (Kahlson and Dixon, 2022). DLD regulates the TCA cycle-related metabolites (Jagtap et al., 2021) and functions together with PDHA1 and PDHB in mitochondrial lipid metabolism (Bhandary and Aguan, 2015). MTF1, known as a conserved metal-binding transcription factor, promotes cell differentiation and protects cells from oxidative stress through responses to copper (Tavera-Montanez et al., 2019). GLS and CDKN2A, as well as MTF1, are composites of the PDH complex, which acts as a sensitizer in copper-induced cell death (Tsvetkov et al., 2022). The protein encoded by CDKN2A is also expressed in a large proportion of patients with UVM and all liver metastases (Russo et al., 2020). These genes interact with each other following copper-increased mitochondrial protein lipoylation, leading to the accumulation of toxic dihydrolipoamide S-acetyltransferase, inhibiting the TCA cycle and leading to cuproptotic cell death (Tang et al., 2022).

Subsequently, according to the Cox analysis, LIAS and DLD had prognostic value for UVM in the TCGA cohort, and these genes were identified as essential regulators of copper-induced cell death (Tsvetkov et al., 2022). The predictability and accuracy of the prognostic signature were confirmed by internal validation including the ROC and calibration plot. Moreover, the AUC for the risk score was 0.814, exceeding those of other clinical factors such as age, gender, pathologic stage, and T-stage, which suggested that the risk score was an influential indicator. It was demonstrated as an independent prognostic factor via multivariate Cox analysis. The prognostic valuation of this signature was further confirmed by an external separate validation cohort from the GEO dataset. These results supported the prognostic value of our CRG-related signature.

Next, we analyzed the DEGs in different risk groups by functional analysis and found that the upregulated DEGs were mostly involved in cell–cell adhesion, extracellular matrix, cilium-based motility, and the peroxisome proliferator-activated receptor (PPAR) signaling pathway. The PPAR signaling pathway is a regulation hub for lipid regulation (Soto-Avellaneda and Morrison, 2020), and it is considered to favor cancer angiogenesis (Wagner and Wagner, 2020). The PPARs susceptibly respond to changes in trace elements such as copper, iron, zinc, and selenium. Thereby, they are critical regulators of lipid homeostasis (Shi et al., 2020). This regulation of lipid is in agreement with the CRGs, which also function in lipid metabolism.

In the GSEA study, the DEGs were involved in GO gene sets of the regulation of SLITs and ROBOs, expression of ROBO receptors, and gene translation. SLITs interact with ROBOs to affect differentiation, cell migration, and cancer initiation (Yang et al., 2010). As a result of SLIT/ROBO signaling, cell-cell adhesion is weakened as the formation of cytoskeletal actin is inhibited and the extracellular matrix disintegrates (Tseng et al., 2015). The GSEA result was in agreement with our GO analysis, which showed that DEGs were enriched in cell adhesion and mobility. GSEA of the hallmark gene set demonstrated that the DEGs were involved in allograft rejection, hypoxia, glycolysis, TNFα signaling via the NF-κB pathway, and interferon-γ responses, suggesting that cuproptosis affects metabolism and immune responses. Based on the aforementioned functional analysis, we speculated that cuproptosis may have the ability to affect the composition of the tumor immune microenvironment. Although in the training cohort, no difference was observed in the total infiltration score between high- and low- risk groups and patients with UVM were predicted to respond poorly to the immune checkpoint therapy, CD8 T cells and exhausted T cells were enriched in the high-risk group. In fact, the low response rates of UVM to ICB may result from its low tumor mutation burden and relatively immune-excluded tumor environment (Kraehenbuehl et al., 2022). In clinical studies, only 0–6% of UVM patients respond to immunotherapy (Heppt et al., 2017), suggesting different patterns of immune infiltration in advanced UVM. Thereby, studies have been carried out to explore the immune cell characterization of UVM. Infiltration of T cells is associated with metastasis and progression of UVM, leading to poor prognosis (Narasimhaiah et al., 2019). Single-cell analysis of metastasized UVM identified that the CD8 T cells and the exhausted subtypes were the main infiltrated immune cells (Durante et al., 2020). These results suggested that cuproptosis may still influence the tumor prognosis via high infiltration of CD8 T cells and exhaust T cells and could be considered as a potential target of future research in UVM.

Finally, to explore the effects of cuproptosis on viability, migration, and invasion of UVM cells *in vitro*, we carried out viability assay, migration assay, and invasiveness transwell assay on UVM cell line M619. After treating the cells with elesclomol- CuCl_2_, the viability of cells decreased, moreover, the ability of migration and invasiveness of UVM cells were also suppressed. Furthermore, we found out that the suppression of viability induced by elesclomol- CuCl_2_ could be reversed by cuproptosis inhibitor TTM. This result confirmed that the inhibitive effect on UVM cells was induced by cuproptosis, suggesting that cuproptosis has a critical role in the development of UVM and it has potential therapeutic value for UVM patients.

This study had several shortcomings. First, the mechanisms of cuproptosis and the functions of CRGs in UVM *in vitro* and *in vivo* are not clear, which deserves further in-depth studies. Second, although we have revealed the prognostic value of the CRGs, prospective clinical studies are still necessary for confirming the usefulness of the CRGs.

In conclusion, our bioinformatic analysis screened eight prognostic CRGs in patients with UVM and constructed a signature and nomogram based on the selected genes, which could predict the OS of patients with medium-to-high accuracy. These genes could be viewed as potential therapeutic targets for UVM following further *in vivo* and *in vitro* studies.

## Data Availability

The datasets presented in this study can be found in online repositories. The names of the repository/repositories and accession number(s) can be found below: https://www.ncbi.nlm.nih.gov/geo/, GSE22138 https://portal.gdc.cancer.gov/, TCGA.

## Supplementary material

Supplementary Figure S1 Cuproptosis related genes.

Supplementary Figure S2 Immune checkpoint blockade therapy results *via* ImmuCellAI.
